# Molecular detection and phylogenetic assessment of six honeybee viruses in *Apis mellifera* L. colonies in Bulgaria

**DOI:** 10.7717/peerj.5077

**Published:** 2018-06-20

**Authors:** Rositsa Shumkova, Boyko Neov, Daniela Sirakova, Ani Georgieva, Dimitar Gadjev, Denitsa Teofanova, Georgi Radoslavov, Maria Bouga, Peter Hristov

**Affiliations:** 1 Agricultural and Stockbreeding Experimental Station, Agricultural Academy, Smolyan, Bulgaria; 2 Department of Animal Diversity and Resources, Institute of Biodiversity and Ecosystem Research, Bulgarian Academy of Sciences, Sofia, Bulgaria; 3 Department of Pathology, Institute of Experimental Morphology, Pathology and Morphology and Anthropology with Museum, Bulgarian Academy of Sciences, Sofia, Bulgaria; 4 Department of Biochemistry, Faculty of Biology, Sofia University “St. Kliment Ohridski”, Sofia, Bulgaria; 5 Laboratory of Agricultural Zoology and Entomology, Agricultural University of Athens, Athens, Greece

**Keywords:** Honey bee viruses, RT-PCR, *Apis mellifera*, Bulgaria

## Abstract

Honey bee colonies suffer from various pathogens, including honey bee viruses. About 24 viruses have been reported so far. However, six of them are considered to cause severe infection which inflicts heavy losses on beekeeping. The aim of this study was to investigate incidence of six honey bee viruses: deformed wing virus (DWV), acute bee paralysis virus (ABPV), chronic bee paralysis virus (CBPV), sacbrood virus (SBV), kashmir bee virus (KBV), and black queen cell virus (BQCV) by a reverse transcription polymerase chain reaction (RT-PCR). A total of 250 adult honey bee samples were obtained from 50 colonies from eight apiaries situated in three different parts of the country (South, North and West Bulgaria). The results showed the highest prevalence of DWV followed by SBV and ABPV, and one case of BQCV. A comparison with homology sequences available in GenBank was performed by phylogenetic analysis, and phylogenetic relationships were discussed in the context of newly described genotypes in the uninvestigated South Eastern region of Europe. In conclusion, the present study has been the first to provide sequencing data and phylogenetics analyses of some honey bee viruses in Bulgaria.

## Introduction

The Western honey bee (*Apis mellifera* L., Hymenoptera: Apidae) is a species of crucial economic, agricultural and environmental importance. Many factors lead to dramatic reduction of bee populations, reduction of bee production, and thus to disruption of the process of pollination of farming crops and wild plants (including certain endemic ones). Among such factors are known diseases with economic significance affecting the bees and their brood, such as bacterial, parasitic and viral (Dainat et al., 2012).

Viral diseases affect honey bee colonies and lead to severe economic losses in the beekeeping industry (Ball & Bailey, 1997; Berthoud et al., 2010; McMahon et al., 2016; Natsopoulou et al., 2017). The most commonly identified honey bee viruses are 30-nm isometric particles with a single-stranded positive RNA (ssRNA) (Tentcheva et al., 2004). The size of the genome of honey bee viruses ranges from 3,700 bp for CBPV (Ribiere, Olivier & Blanchard, 2010) but usually from 8,000 up to 10,000 bp (Tantillo et al., 2015). The genome of ABPV, BQCV, and KBV viruses consists of two open reading frames (ORFs) in the 5′ and 3′ end directions, encoding two polyproteins, while iflaviruses, like Sacbrood virus, and Deformed wing virus, DWV (including viruses Varroa destructor virus 1, VDV1, Kakugo virus strains have a single ORF. The first ORF region encodes non-structural proteins involved in virus replication and processing while the 3′ flanking region encodes the structural proteins found in the viral particle (Tantillo et al., 2015). Usually, different protocols based on 5′ and 3′ flanking ORF regions (ORF1 and ORF2) have been used for virus detection (Tentcheva et al., 2004; Maori et al., 2007). Due to this reason, most investigations use different sequencing sets concerning phylogenetic analyses and geographical dispersal.

The SBV and DWV are assigned to the Family *Iflaviridae,* Genus *Iflavirus*, while ABPV, KBV and BQCV are members of the Family *Dicistroviridae*, Genus *Cripavirus* (Mayo, 2002). Up to now, CBPV has been the only virus which has not been classified yet because there is evidence that this virus is distinct from the other ones with respect to particle symmetry and size, and genome composition and organization (Ribiere, Olivier & Blanchard, 2010). All of those viruses are widely dispersed worldwide (Ellis & Munn, 2005; Reynaldi et al., 2010; Reynaldi et al., 2011).

Most investigations based on phylogenetic analysis have revealed multiple, i.e., ABPV and DWV, or endemic East Asian and Australian (Pacific region), and Indian origin (BQCV and SBV) of most described honey bee viruses as well as their introduction in the West direction toward Europe and worldwide (Tapaszti et al., 2009; Bakonyi et al., 2002; Yang et al., 2013).

Despite the importance of viral diseases on honey bee colonies health, the sequencing and phylogenetic data related to the origin and spreading of viruses on the Balkan Peninsula are scarce (Hatjina et al., 2011; Simeunović et al., 2014).

Bulgaria as a part of the Balkans and a bordering region between the Middle East and Europe is an interesting point for understanding the dissemination and introduction of honey bee viruses. Therefore, in this study, we have reported the first survey of six honey bee viruses (DWV, ABPV, CBPV, SBV, KBV and BQCV) in honey bee colonies in Bulgaria. A phylogenetic analysis was performed to reveal the geographic distribution of different virus strains.

## Materials and Methods

### Honey bee sampling

Samples of adult worker bees were collected from 50 colonies originating from eight apiaries in three locations in Bulgaria: Rousse district (North Bulgaria, *n* = 20), Sofia district (West Bulgaria, *n* = 20), and Smolyan district (South Bulgaria, *n* = 10), (Table 1), during spring and the beginning of summer (May–June 2017). In the two first regions the introduced honey bees are reared (*A. m. carnica, A. m. ligustica* and *A. m. macedonica*), while in the last region a native Bulgarian honey bee named *A. m. rodopica* exists. There was no bias concerning honey bee samples collection.

**Table 1:** Distribution of DWV, SBV, ABPV, BQCV, CBPV, and KBV viruses in three different regions in Bulgaria (from 50 apiaries).

Region^a^	No. of hives	DWV	SBV	ABPV	CBPV	BQCV	KBV
Smolyan (SB)	10	+ +^c^	−^b^	−	−	−	−
Sofia (WB)	20	+ + +	+ + + + + +	−	−	+	−
Rousse (NB)	20	+ + + + +	−	+ +	−	−	−
No. (%) of positive colonies		10 (20)	6 (12)	2 (4)	0	1 (2)	0

**Notes.**

a SB, South Bulgaria; WB, West Bulgaria; NB, North Bulgaria.

b (+), Positive; (−), Negative.

c number of detected viruses in each studied region.

While in the mountainous part of the country (Smolyan district) the honey and pollen diet is very diverse (consisting mainly of meadow flora), in the flat regions (Rousse and Sofia district) there is monofloral honey and pollen (rapeseed, sunflower, lime tree, etc.). The apiary density in the mountainous part of the country is from 5 to 10 km away, while in the flat regions of the country this distance is rather shorter (up to 1 km). In the Rhodope mountains, apiaries consist of 10 to 25 hives, while in flat regions the number of hives per apiary is up to 100.

The presence of the Varroa mite was not checked for each colony at the time of sampling, but all honey bee colonies are treated with acaricides during the autumn period.

Different subspecies like *A. m. carnica*, *A. m. ligustica* and *A. m. macedonica* have been imported from Central Europe and reared mainly in the flat regions in the country. In the Rhodope mountains, the local honey bees (*A. m. rodopica*) are undergoing selection control as part of the national biodiversity conservation. Beekeepers are encouraged to rear only the local honey bee, and crossbreeding with other races is not allowed.

From each colony, five adult bees (visually healthy, asymptomatic for bacterial, fungal and viral clinical signs) were collected individually, following the method described by Chen et al. (2004). There were no bees with clinical symptoms in these hives; the collected adults were typical. The obtained samples were put in a cooler bag and immediately sent to the laboratory, where they were frozen at −20 °C.

The experimental design had been carried out under permissions and the guidelines of the Bulgarian Academy of Sciences and the Bulgarian Ministry of Environment and Waters (no. 627/30.03.2015).

### Total RNA extraction and RT-PCR amplification

The frozen samples were crushed in a mortar and were homogenized in an RL lysis buffer (GeneMATRIX Universal RNA Purification Kit, Cat. No. E3598; EURx Ltd., Gdansk, Poland). After homogenization, the samples were centrifuged for 3 min at 15,000× g to remove unhomogenized particles. An aliquot of supernatant was used for the extraction of total RNA according to the manufacturer’s recommendations. The quality of the extracted total RNA was checked by electrophoresis and spectrophotometry. An average of 2 µg of the total RNA was used for copy DNA (cDNA) synthesis using Oligo(dT)_20_ primers (NG dART RT-PCR kit, E0802; EURx Ltd., Gdansk, Poland) according to the manufacturer’s instructions.

The PCR mixture contained 25 µL of NZYTaq 2× Colourless Master Mix (Cat. No. MB04002; Nzytech, Lisboa, Portugal), 0.4 µM of each virus specific primer (FOR/REV), 1 µL of template cDNA in a total volume of 50 µL. All RT-PCR amplifications were carried out using a Little Genius thermocycler (BIOER Technology Co., Ltd., Hangzhou, China) under the following conditions: initial denaturation at 94 °C for 5 min; 35 cycles (denaturation at 94 °C for 30 s; primer annealing at 56 °C for 30 s; extension at 72 °C for 1 min) and final extension at 72 °C for 10 min. The PCR products were visualized on 1% agarose gel with GreenSafe Premium (Cat. No. MB13201; Nzytech, Lisboa, Portugal). The fragment size was determined using Gene-Ruler™ 100 bp Ladder Plus (Cat. No. SM0323; Thermo Fisher Scientific Inc., Waltham, MA, USA). The primers used for detection of the viruses (DWV, ABPV, CBPV, SBV, KBV and BQCV) had been described by Tentcheva et al. (2004). The successfully amplified products were purified by a PCR purification kit (Gene Matrix, PCR clean-up kit, EURx, Poland) and sequenced in both directions by a PlateSeq kit (Eurofins Genomics Ebersberg, Gdansk, Germany).

### Statistical and phylogenetic analysis

All obtained DNA sequences were manually edited and aligned with reference complete viral genomes (ABPV, Acc. No. AF150629, (Govan et al., 2000); DWV, Acc. No. NC_004830, (Lanzi et al., 2006); SBV, Acc. No. NC_002066, (Ghosh et al., 1999); BQCV Acc. No. NC_003784, (Leat et al., 2000)) by using the MEGA7 program (Kumar, Stecher & Tamura, 2016). The obtained sequences (DWV—388 bp, SBV—417 bp, ABPV—435 bp and BQCV—486 bp) were deposited in the GenBank database National Biotechnology Information Center (NCBI) under accession numbers MG599458–MG599464 and MG649495–MG649502. All obtained sequences covered the 3′ end of the ORF region of the viral genome. This part of the viral genome sequences was chosen for analysis based on the availability of most similar sequences as well as complete viral genomes of other countries’ viral isolates. After retrieving appropriate sequences from GenBank, all sequences were aligned using MUSCLE (Edgar, 2004), and then the best-fit substitution model was selected for constructing each viral phylogeny. The phylogenetic tree was constructed using the maximum likelihood method and a bootstrap value of 10,000 replicates with the MEGA7 program (Kumar, Stecher & Tamura, 2016) in each case. The phylogenetic trees were visualized by using FigTree v 1.4.3 (http://tree.bio.ed.ac.uk/software/figtree/).

The 95% confidence intervals for all detected viruses were calculated by using the Confidence Interval of a Proportion (http://vassarstats.net/prop1.html), (Newcombe, 1998).

## Results

### Virus frequencies in honey bee colonies

The data for RT-PCR detection of six bee viruses in the 50 hives are shown in Table 1. Except for CBPV and KBV, which were not detected, the other four viruses were found. DWV was found to be with the highest frequencies (10.20%, the 95% confidence interval (CI) was between 11 to 34). It was observed in all regions of the country—South Bulgaria (Smolyan; 2.20%, 95% CI of a proportion was 3.5% to 56%), West Bulgaria (Sofia 3.15%, 95% CI of a proportion was calculated to be 5% to 36%) and North Bulgaria (Rousse; 5.25%, 95% CI of a proportion was 11% to 47%). The fact that this virus is present in honey bee colonies both in the flat and in the mountainous parts of the country shows that there are no significant differences between the different regions. SBV was the second most frequent one of those studied (6.12%, 95% CI of a proportion 11 to 47). This virus was found only in West Bulgaria (Sofia; 6.30%) and nowhere in the other regions. In contrast to SBV, ABPV was detected only in North Bulgaria (2.10%), but with a low frequency (2.4%, 95% CI of a proportion was 1 to 14) in all 50 investigated colonies. This virus appears to show prevalence in the flat regions of the country. BQCV was with the lowest frequency, recorded only in West Bulgaria (Sofia; 1.5%, 95% CI of a proportion was 2.5 to 27) or only 2% from all samples.

## Discussion

### DWV

DWV is one of the larger widely distributed honey bee viruses worldwide (Allen & Ball, 1996). The main vector for this virus is *Varroa destructor*, which might explain why this virus was not detected in Varroa-free colonies in Australia (Roberts, Anderson & Durr, 2017) and was present in the honey bees in Varroa—free Hawaiian and Scottish islands at much lower levels compared to the Varroa-infested regions (Martin et al., 2012; Ryabov et al., 2014). Our results have demonstrated that this virus is present in all investigated regions in Bulgaria (Table 1). Seven samples were successfully amplified and sequenced (GenBank Acc. No. MG599458–MG599464). In the Balkans, this virus was found in Greece (Hatjina et al., 2011; Meixner et al., 2014), Serbia (Simeunović et al., 2014), Croatia, Bulgaria and Macedonia (Meixner et al., 2014). In these investigations, only molecular detection of DWV was performed by real-time RT-PCR, but no sequence and phylogenetic analyses were carried out. The incidence of DWV infection in our Bulgarian samples was lower compared to that in other European regions, including the Balkans where the DWV infection rate was over 90% (Tentcheva et al., 2004; Baker & Schroeder, 2008).

DWV includes three closely related strains, which differ in terms of their pathogenic effect and genetic diversity (Brettell et al., 2017): type A, which includes DWV itself (Lanzi et al., 2006) and Kakugo virus (KV), type B, which is also known as Varroa destructor virus-1 (VDV-1) (Ongus et al., 2004), and a distinct type C, (Mordecai et al., 2016).

**Figure 1:**
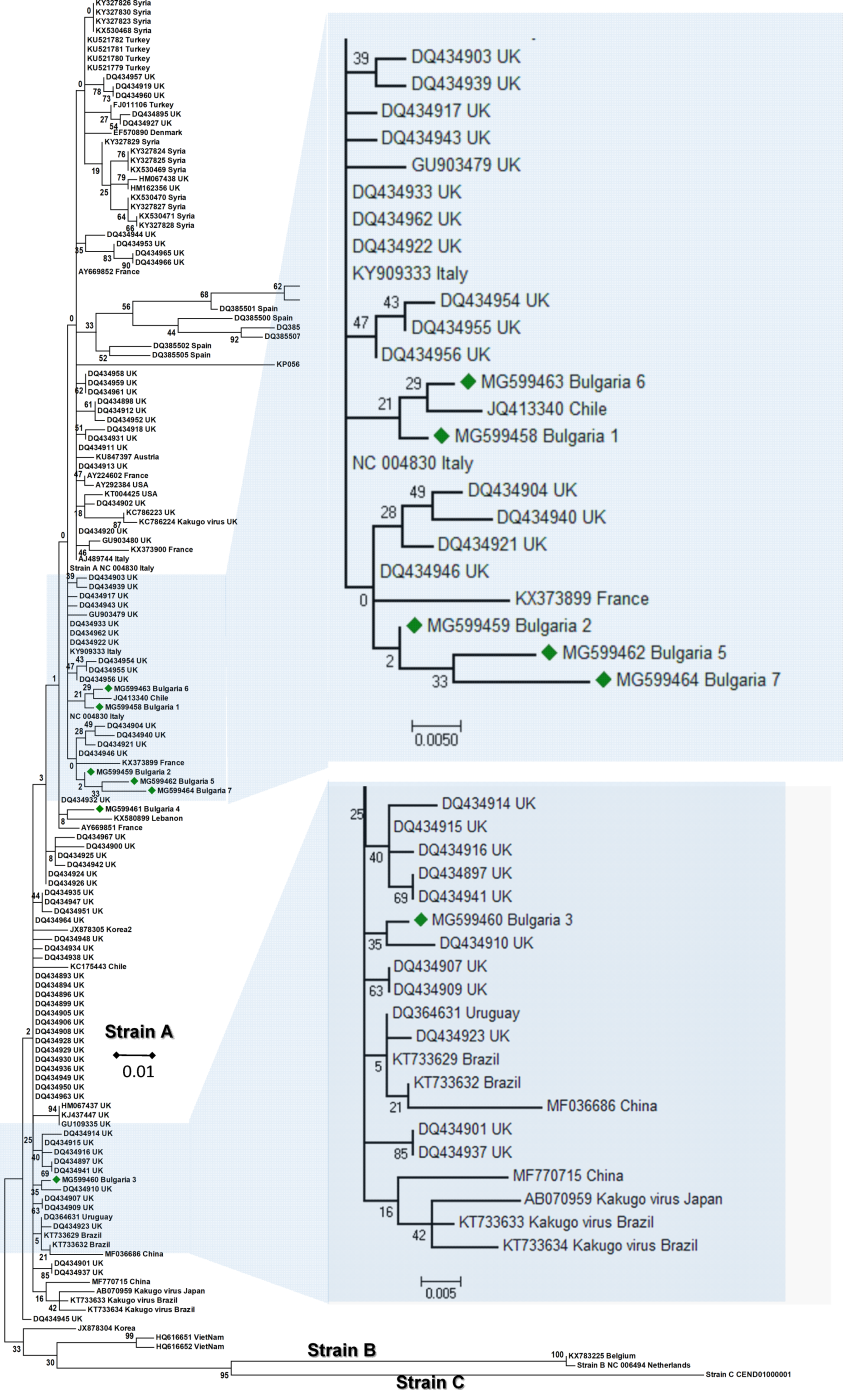
Molecular phylogenetic analysis of deforming wing virus (DWV) isolates from Bulgaria and other countries. The phylogenetic tree based on alignment of the partial (388 bp) protein coding region of DWV (8594 nd to 8960 nd according to Ref. seq. Acc. no. NC_004830). Each isolate is indicated by country of isolation and GenBank accession number. Bulgarian isolates identified in this study are represented by green diamonds. The consensus maximum-likelihood phylogenetic tree was constructed using appropriate models for each virus and 10,000 bootstraps.

The phylogenetic analysis of the Bulgarian DWV sequences showed that they all belonged to type A DWV. From all seven obtained sequences, six split in numerous different branches (Fig. 1). The heterogeneity of our samples was calculated to be 0.021 (Kumar, Stecher & Tamura, 2016). The Bulgarian virus sequences was grouped with six UK branches (Baker & Schroeder, 2008), two branches in Turkish (Acc. No. KU521781–KU521779), and three in Spanish isolates (Acc. No. DQ385499, DQ385501, DQ385502) (Fig. 1). The European DWV strains formed numerous different genetic clades with no clear regional distribution. This suggests that DWV strains dispersal has occurred in all Eurasia (Fujiyuki et al., 2004; Lanzi et al., 2006; Tentcheva et al., 2004; Baker & Schroeder, 2008; Moore et al., 2011; Reddy et al., 2013; Wang et al., 2013; Carrillo-Tripp et al., 2016; Lamp et al., 2016). The phylogenetic analysis of DWV based on different genome regions (encoding the capsid protein and the part of RNA dependent-RNA polymerase) has also revealed numerous different geographically determined clades (US, Japan, China), but not European ones (Yang et al., 2013).

It may be concluded that the Bulgarian DWV sequences shared a common origin with the Turkish virus strains as well as with those in other Mediterranean countries.

### SBV

SBV can infect either larvae or adult honeybees, with a higher sensibility of larvae to the infection. SBV primarily affects the brood of the honey bees, whichcauses significant morphological alterations resulting in larval death (Berenyi et al., 2006). We found six cases of SBV infection in our samples (12%), (Table 1). Five of them were successfully sequenced and deposited in GenBank under Acc. No. MG649495–MG649499. To date, in the Balkans, this virus has been detected once in Greece (Hatjina et al., 2011).

**Figure 2:**
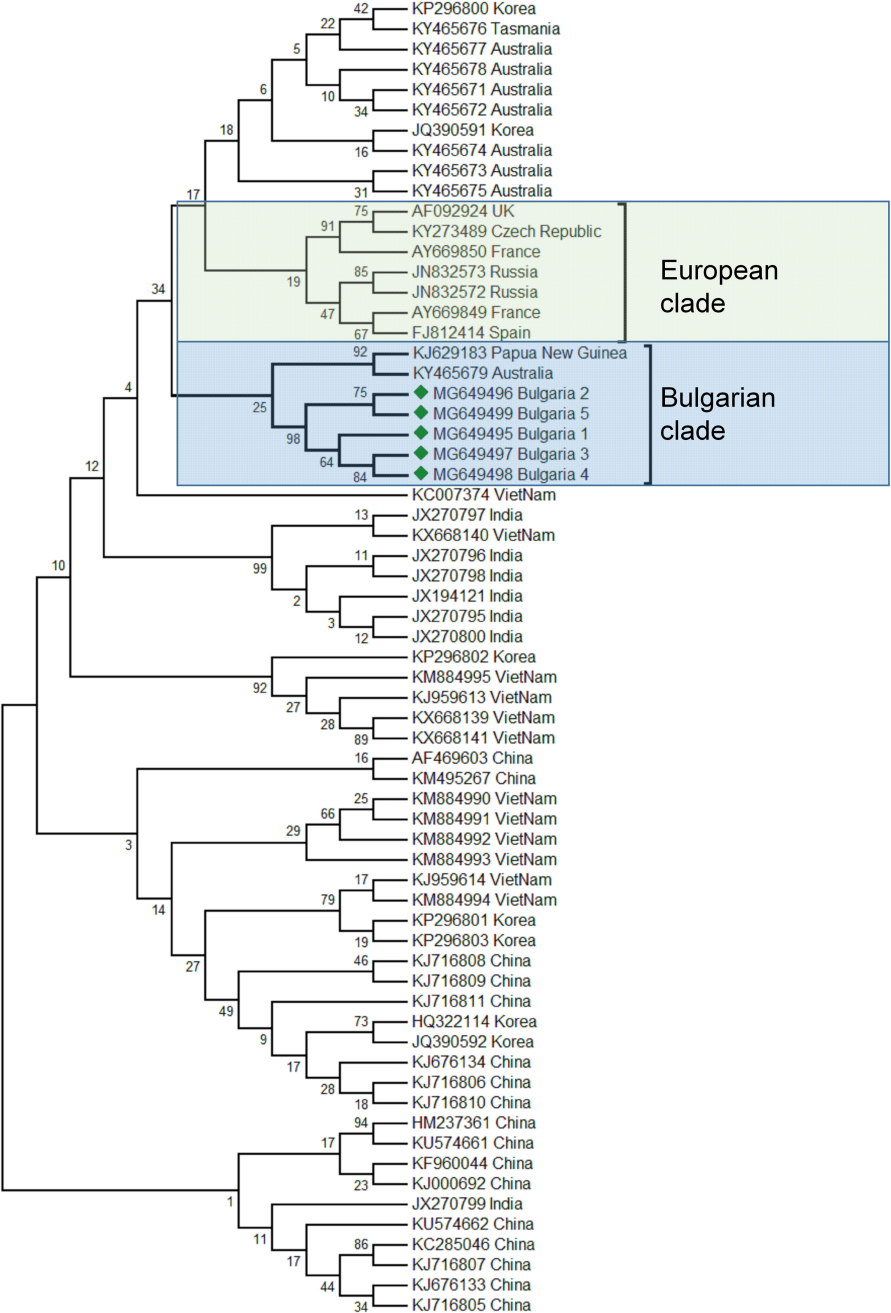
Molecular phylogenetic analysis of Sacbroad virus (SBV) isolates from Bulgaria and other countries. The phylogenetic tree based on alignment of the partial (417 bp) protein coding region of SBV (7760 bp to 8176 bp according to Ref. seq. Acc. no. NC_002066). Each isolate is indicated by country of isolation and GenBank accession number. Bulgarian isolates identified in this study are represented by green diamonds. The consensus maximum-likelihood phylogenetic tree was constructed using appropriate models for each virus and 10,000 bootstraps.

The phylogenetic analysis of the Bulgarian samples showed that they can be grouped with the samples from Papua (New Guinea) and Australia (Acc. No. KJ629183, (Roberts & Anderson, 2014); Acc. No. KY465679, (Roberts, Anderson & Durr, 2017)). The estimated average divergence within this cluster was calculated to be 0.163 (Kumar, Stecher & Tamura, 2016). SBV from Europe formed a different clade including available sequences from the West to the East (Fig. 2) (Ghosh et al., 1999; Tentcheva et al., 2004; Lomakina & Batuev, 2012). The genetic distance between the Bulgarian and the European clade was calculated to be 0.216 (Kumar, Stecher & Tamura, 2016). The European clade analyzed by 5′ ORF1 also includes additional data (Grabensteiner et al., 2001). Based on this sequence data, the European clade has possibly originated from Nepal, while the Bulgarian clade is potentially of Australian origin. The US clade of SBV otherwise has a high homology and possible introduction with Chinese virus strains (Yang et al., 2013).

Taking together phylogenetic data for SBV worldwide showed high diversity split in different clades from the Pacific region (Australia, China, South Korea, India, Vietnam, etc.) (Roberts, Anderson & Durr, 2017; Ma et al., 2011; Xia, Zhou & Wei, 2015; Reddy et al., 2016; Nguyen & Le, 2013).

The phylogenetic analysis of the Balkan SBV revealed new European genotypes, introduced in a different way in Europe.

### BQCV

**Figure 3:**
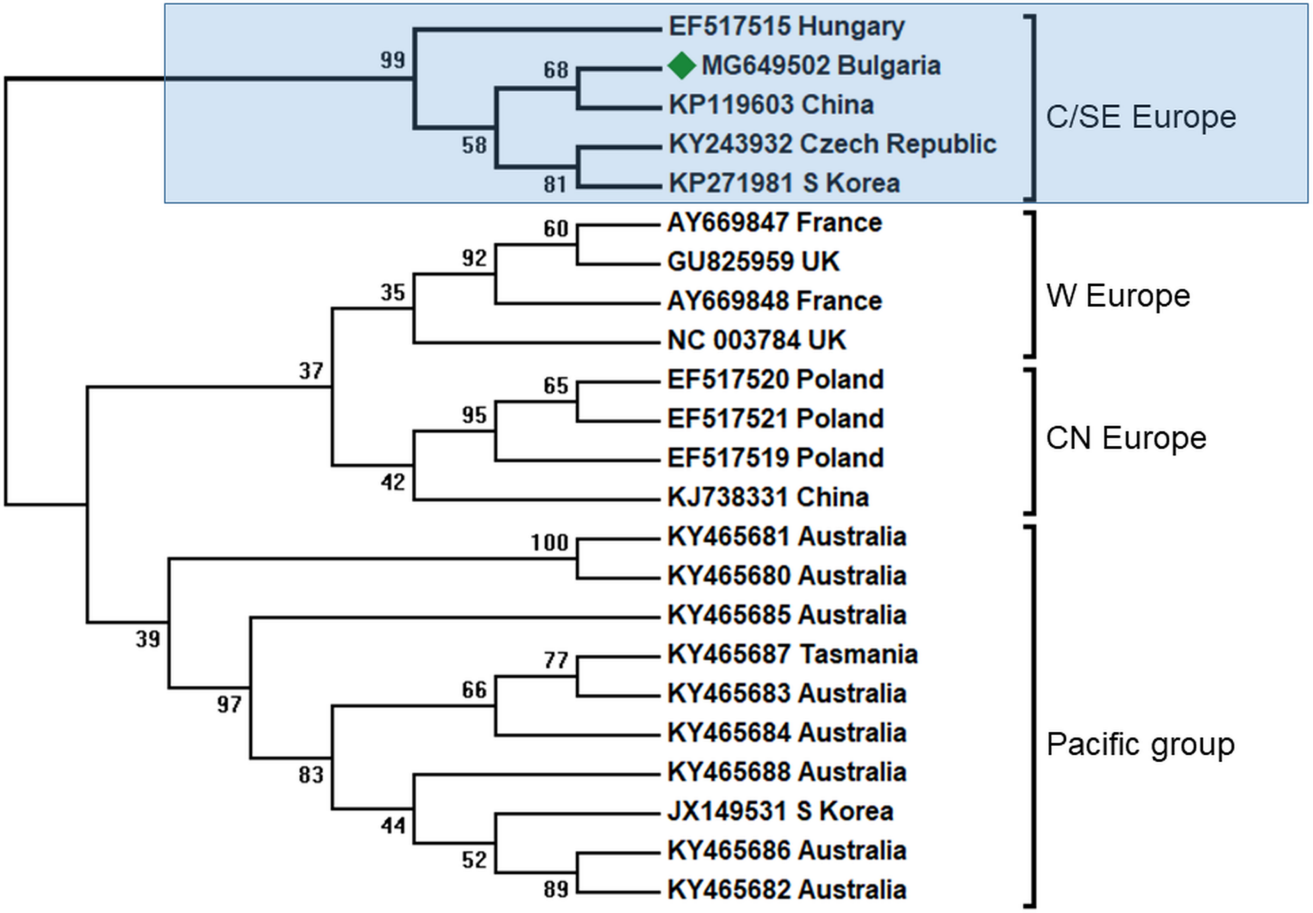
Molecular phylogenetic analysis of black queen cell virus (BQCV) isolates from Bulgaria and other countries. The phylogenetic tree based on alignment of the partial (486 bp) protein coding region of BQCV (4,603 bp to 5,088 bp according to Ref. seq. Acc. no. NC_003784). Each isolate is indicated by country of isolation and GenBank accession number. Bulgarian isolate identified in this study is represented by green diamond. C/SE Europe—Central/South-Eastern Europe; W Europe—Western Europe; CN Europe—Central-Northern Europe. The consensus maximum-likelihood phylogenetic tree was constructed using appropriate models for each virus and 10,000 bootstraps.

BQCV is one of the most abundant honey bee viruses, about 80% after DWV and SBV (Tapaszti et al., 2009). Previous studies from the Balkan region had shown that in Greece honey bee colony infection was also the most abundant in all investigated apiaries (Hatjina et al., 2011). In our study, we found only one case of infection (Table 1). Phylogenetic analysis of the obtained sequenced fragment (Acc. No. MG649502) showed the highest homology with European BQCV strains from the Czech Republic (95%) and Hungary (93%) (Tapaszti et al., 2009; Spurny et al., 2017), (Fig. 3). These data support three centers of virus diversity in Europe—Central and South-Eastern (CSE cluster), Central-North (CN cluster, Poland) and West Europe (WE cluster, France and UK) (Tentcheva et al., 2004; Tapaszti et al., 2009; Roberts, Anderson & Durr, 2017; Spurny et al., 2017). The last two clusters—CN and WE are split from the CSE clade together with the Pacific group (Australia, Tasmania, South Africa and South Korea) (Roberts, Anderson & Durr, 2017). The results for the 3′ ORF flanking region were also supported with virus phylogeny and diversity based on the more variable ORF1 region (Roberts, Anderson & Durr, 2017). Considering this data, the CSE BQCV clade also includes regions in Austria and Germany (Tapaszti et al., 2009; Reddy et al., 2013).

In all clusters there exist different BQCV genotypes from China and Korea. This may be explained by the fact that there are at least three virus strains that infected honey bees in Europe imported from Central Asia (Fig. 3).

It is obvious that the genetic diversity of viruses in Europe is supported by the geographic spreading in Europe, possibly by independent transfer of infection without co-infections. Our data enlarge the geographic spreading of CSE BQCV strains in South-Eastern direction.

### ABPV

**Figure 4:**
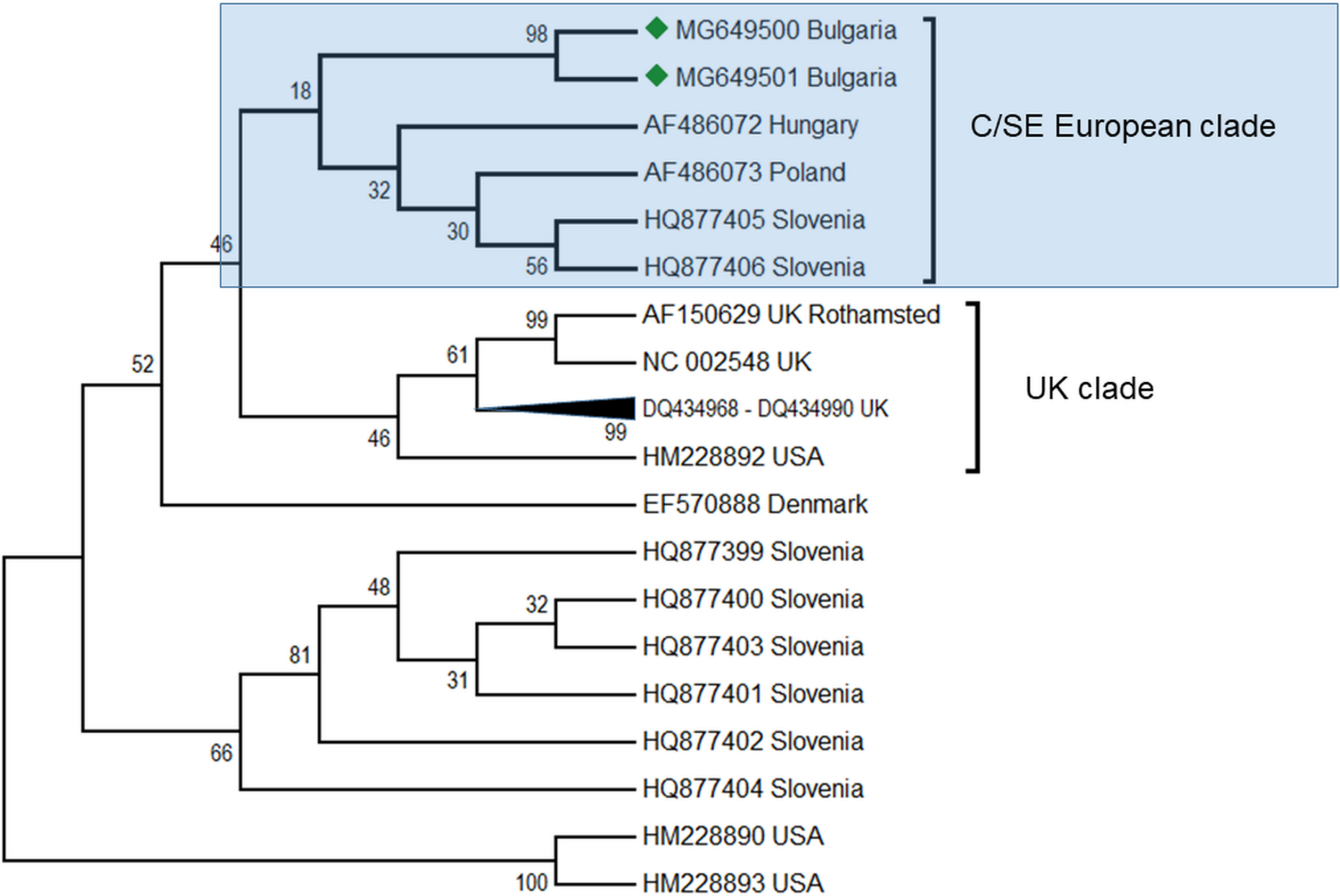
Molecular phylogenetic analysis of acute bee paralysis virus (ABPV) isolates from Bulgaria and other countries. The phylogenetic tree based on alignment of the partial (435 bp) protein coding region of ABPV (5,273 bp to 5,707 bp according to Ref. seq. Acc. no. AF150629). Each isolate is indicated by country of isolation and GenBank accession number. Bulgarian isolates identified in this study are represented by green diamonds. The consensus maximum-likelihood phylogenetic tree was constructed using appropriate models for each virus and 10,000 bootstraps.

ABPV is also frequently found in honey bee colonies. Apparently, this virus plays a role in cases of a sudden collapse of the honey bee and is transmitted by the parasitic mite *V. destructor* (Bakonyi et al., 2002). In the Balkans, this virus was found together with DWV in Greece (Hatjina et al., 2011; Meixner et al., 2014), Serbia (Simeunović et al., 2014) as well as in Croatia, Bulgaria and Macedonia (Meixner et al., 2014) as described above. In these studies the intensity of the infection varied from 50 to 100%. The presence of this virus was also observed together with a mixed infection with DWV. In our study, this virus was found in two honey bee colonies (2, 4%) (Table 1) and sequencing fragments were deposited in GenBank under Acc. No. MG649500 –MG649501. The phylogenetic analysis revealed high homology (97%) with ABPV genotypes from Hungary (Fig. 4), (Bakonyi et al., 2002) and from a clade with Slovenian viruses (Jamnikar Ciglenecki & Toplak, 2013). The estimated average divergence within this clade was calculated to be 0.018 (Kumar, Stecher & Tamura, 2016). Up to now, genetic analyses of ABPV in Europe have clearly distinguished three main clades (Bakonyi et al., 2002). There are two clades concerning Central Europe that are split into two branches—Hungary and Austria-Germany, and a third one which is more distinct in the UK. In Poland, ABPV shows mixed infection between these clades (Bakonyi et al., 2002). The UK clade is closely related to the US strains (Govan et al., 2000; Li et al., 2010; Baker & Schroeder, 2008). To date, there have been no data about ABPV outside Europe and North America (the USA and Canada) despite the presence of the main transmission agent *V. destructor* (Yang et al., 2013). This virus is also not detected in Australia due to the absence of *Varroa* (Roberts, Anderson & Durr, 2017).

Our findings have enlarged the existing data on the geographic spreading of ABPV up to South-Eastern Europe, where this virus was detected for the first time.

We suggest that the different levels of virus infections may be partly explained by the climatic conditions in the mountainous and the plain regions. The Rhodope Mountains climate is rather colder than the other two investigated regions and, possibly, with a lower rate of infection with Varroa mites. This conclusion was supported by other studies which have observed the lowest infection with Varroa, Nosema and Virus in the regions with colder climate in Europe (Meixner et al., 2014).

## Conclusion

The present study revealed the first sequenced data reported for the Balkan Peninsula and phylogenetic data about geographic distribution and genetic diversity including new data sets in the Balkans. The phylogenetic analysis of ABPV and BQCV has shown a close relation to Central-European virus strains that differ from West-European and Polish genotypes. The Bulgarian samples of SBV seem to form a new distinct clade within the European strains with a higher homology to the isolates from the Pacific region. The multiple genotypes of DWV are present in Bulgaria and are most closely related to the Mediterranean and Turkish DWV strains.

## Additional Information and Declarations

### Field Study Permissions

The following information was supplied relating to field study approvals (i.e., approving body and any reference numbers): The experimental design was carried out with permission and under the guidelines of the Bulgarian Academy of Sciences and the Bulgarian Ministry of Environment and Waters (no. 627/30.03.2015).

### Data Availability

The following information was supplied regarding data availability: The obtained sequences (DWV - 388 bp, SBV - 417 bp, ABPV - 435 bp and BQCV - 486 bp) were deposited in the GenBank database National Biotechnology Information Center (NCBI) under accession numbers MG599458–MG599464 and MG649495–MG649502.
